# Statistical Physics for Medical Diagnostics: Learning, Inference, and Optimization Algorithms

**DOI:** 10.3390/diagnostics10110972

**Published:** 2020-11-19

**Authors:** Abolfazl Ramezanpour, Andrew L. Beam, Jonathan H. Chen, Alireza Mashaghi

**Affiliations:** 1 Leiden Academic Centre for Drug Research, Faculty of Mathematics and Natural Sciences, Leiden University, 2333CC Leiden, The Netherlands; a.ramezanpour@lacdr.leidenuniv.nl; 2 Department of Physics, School of Sciences, Shiraz University, 71454 Shiraz, Iran; 3 Department of Biomedical Informatics, Harvard Medical School, Boston, MA 02115, USA; Andrew_Beam@hms.harvard.edu; 4 Department of Epidemiology, Harvard T.H. Chan School of Public Health, Boston, MA 02115, USA; 5 Department of Newborn Medicine, Brigham and Women’s Hospital, Boston, MA 02115, USA; 6 Biomedical Informatics, Stanford University School of Medicine, Stanford, CA 94305-5101, USA; jonc101@stanford.edu; 7 Department of Medicine, Stanford University School of Medicine, Stanford, CA 94305-5101, USA

**Keywords:** diagnostic process, statistical physics, disease progression

## Abstract

It is widely believed that cooperation between clinicians and machines may address many of the decisional fragilities intrinsic to current medical practice. However, the realization of this potential will require more precise definitions of disease states as well as their dynamics and interactions. A careful probabilistic examination of symptoms and signs, including the molecular profiles of the relevant biochemical networks, will often be required for building an unbiased and efficient diagnostic approach. Analogous problems have been studied for years by physicists extracting macroscopic states of various physical systems by examining microscopic elements and their interactions. These valuable experiences are now being extended to the medical field. From this perspective, we discuss how recent developments in statistical physics, machine learning and inference algorithms are coming together to improve current medical diagnostic approaches.

## 1. Introduction

The use of machine intelligence may transform how physicians diagnose and treat their patients. Artificial intelligence (AI) has been successfully applied to extract “signs” or “biomarkers” from complex measured data. This approach has provided significant assistance to the diagnosis/ classification of various diseases using phenotypic and genotypic information or medical images. Remarkably, deep learning models have achieved physician-level accuracy in a broad variety of diagnostic tasks, including distinguishing moles from melanomas, identifying diabetic retinopathy, detecting breast lesions in mammograms, and performing spinal analysis based on magnetic resonance imaging [1,2,3,4,5]. However, a key limitation across studies that have compared human and algorithmic performance has been a lack of clinical context (e.g., medical history and laboratory findings), which is critically important for solving many diagnostic challenges. Rapid progress in omics technology has led to the availability of large sets of medical data, providing detailed biochemical context. Omics measurements can provide the concentrations of thousands of proteins, metabolites and RNA molecules based on the analysis of small volumes of body fluid. Moreover, electronic health records are rapidly becoming ubiquitous, thereby making the medical transactions of millions of patients available. Finally, large amounts of data from healthy individuals are becoming available through prospective, population-based cohort studies. The application of computational approaches to such complex data sources makes it possible to generate insights that would be impractical to extract through manual human review alone. This opens up major opportunities for the application of AI technology in medical diagnostics; however, new conceptual advancements are needed before these possibilities can be explored.

Decision making lies at the heart of medicine and is often a tedious task. There are many practical challenges in clinical practice and decision making—diagnosis and prognosis are among those that may most naturally benefit from data-driven approaches. A recent report by the National Academy of Medicine revealed many diagnostic errors that could be mitigated by appropriate measurement and support. Diagnostic strategies are typically available in the form of clinical algorithms and flow charts that define the sequence of actions to be taken to reach a diagnosis. A diagnosis itself is typically made based on consensus diagnostic criteria [6,7,8,9]. For example, various algorithms exist for approaching a patient with abdominal pain, which can be a manifestation of autoimmune diseases such as systemic lupus erythematosus (SLE), among others. Despite differences in diagnostic approaches to this symptom, consensus diagnostic criteria for SLE are available. Overall, however, we lack a solid conceptual framework for medical diagnostics, particularly in the early stages of disease development and in the presence of multiple interacting diseases. As a consequence, there is no consensus on the diagnostic flow charts available today, and clinicians differ widely in their approaches to patients.

Diagnostic problems are sometimes difficult to solve, partly due to a lack of critical information about disease mechanisms and manifestations, uncertainties in some observations, and often-overlooked disease–disease interactions. In essence, however, a diagnostic problem asks simply for the most probable disease hypothesis given an initial set of observed signs (symptoms as well as clinical and laboratory findings) along with some prior knowledge about the patient. To be specific, let us consider the biochemical reaction network of an organism as the system under study [10,11,12]. Here, the activities or concentrations of the molecular species can be taken as the system signs, and deviations from the healthy network structure and the associated reaction rates can be interpreted as the system defects. The problem here is to uncover the set of involved defects from the observed molecular concentrations for a given number of species. This is a computationally hard problem, with a computation time that grows exponentially with the number of signs and diseases [13,14]. Already, the numerical simulation of such a system is computationally expensive for practically interesting reaction networks due to the presence of many dynamical time scales (reaction rates) in the system [15,16]. It is known that stochastic reaction networks can perform reliable Turing-universal computations, and simulating such systems in general cannot be an easy task (see no-free-lunch theorems) [17,18,19]. Basically, the latter problem is equivalent to the problem of inference from a probabilistic model of many interacting sign and disease variables. In practice, for the study of these probabilistic models, we resort to simplifying assumptions, such as the causal independence of diseases and the conditional independence of signs (given a disease hypothesis) [20,21,22,23]. Another common assumption is that a “single disease” underlies a patient’s symptoms/signs; however, this “simplifying” assumption may sometimes complicate the diagnostic problem (some observed signs will be deemed as uninterpretable). For example, a patient with a fever, cough, and diarrhea may clinically present as a single diagnosis of viral infection, but these symptoms could instead be due to a combination of multiple factors, such as cancer with infectious complications. Age-related diseases are another class of co-emerging health problems.

**Figure 1:**
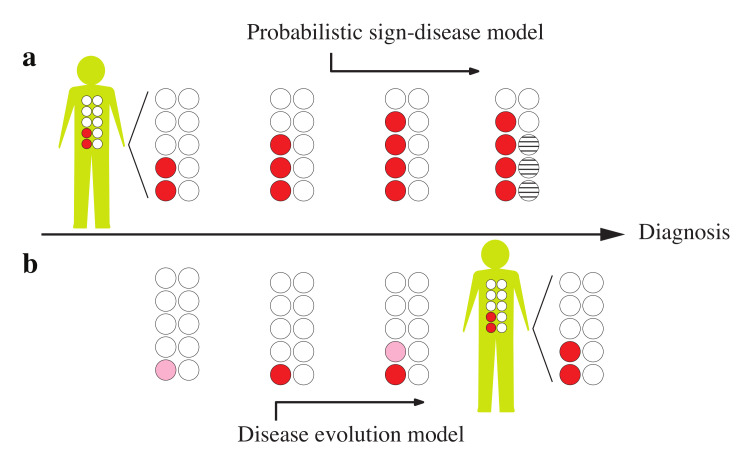
Uncovering (learning) macroscopic features (diagnosis) from microscopic sign variables: (**a**) using a powerful probabilistic model for a selective observation of additional signs and a careful anticipation of a few other sign values by simulating the diagnostic process; (**b**) using a microscopic model of disease evolution to estimate the likelihood of a disease hypothesis from the history (dynamics) of the observed signs. Here, empty circles indicate the unobserved signs, the filled circles are the observed signs with possibly different levels of activity, and the dashed circles show the anticipated sign values.

The diagnostic problem is more important to address in the early stages of a disease, when the amount and quality of medical evidence are insufficient to reach a definite diagnosis by conventional methods. This early diagnosis should of course be efficient and accurate to have the most sensitivity and specificity with the minimum possible cost and in an acceptable time. It is also important to know how the diagnostic accuracy changes with disease development to understand the trade-off between the timing and accuracy of the diagnosis. Note that an early and precise diagnosis also requires an accurate characterization of disease states, and an understanding of the mechanisms of disease development (dynamics) and the way in which one disease influences others (disease interactions). This, along with the acquisition of good statistical data, can allow us to construct more accurate diagnostic models and algorithms for uncovering hidden disease patterns in the early stages of their progress (Figure 1).

The concepts and tools of statistical physics, computer science, and graph theory have proven very helpful in the study of similar problems. Examples include the reconstruction of biological models (e.g., neural networks) from observed statistical data [24,25,26,27,28], physically inspired error correction and compressed sensing algorithms [29,30], and a complex network approach to biology and medicine [31,32,33,34]. Statistical physics has been widely used to extract macroscopic properties of many interacting elements from their microscopic models. This process is largely analogous to the extraction of disease states from a patient’s signs and symptoms (see Table 1); however, no similar conceptual framework has yet been applied to medical diagnostics. On the other hand, the construction of good probabilistic models and the extraction of accurate information from such models using efficient inference and optimization algorithms play a critical role in the study of diagnostic problems [35,36,37,38]. An interdisciplinary perspective is necessary here to go beyond the conventional diagnostic models and strategies to deal with the above problems. In what follows, we discuss opportunities and challenges that lie ahead.

## 2. Disease Definition and Classification

In the classical picture of a diagnostic problem, we usually assume that we have a given set of defined signs/symptoms S and a given set of diseases D. Note that a symptom could refer to an objective indicator, e.g., blood pressure, whereas a sign could refer to something that only the patient is able to assess, such as pain intensity. The sign and disease values are denoted by $S=\{S_{i}:i=1,\cdots ,N\}$ and $D=\{D_{a}:a=1,\cdots ,M\}$, respectively. For simplicity, in the following it is assumed that $S_{i}=\pm 1$ and $D_{a}=0,1$. This is obviously very useful for a supervised learning algorithm using (deep) neural networks for disease classification [39,40,41]. The signs are usually the input variables for a multilayer feed forward network and the diseases are coded in the states of the output variables. On the other hand, one may consider a recurrent neural network to learn the related sign–disease patterns from the observations, for example, by using the Hebb’s rule. The well developed statistical physics methods have been used in both the cases to provide useful insights about the quality of solutions and the performance of the learning algorithms [42,43,44,45,46,47].

In practice, however, the boundary between signs and diseases is not always clear. For instance, addiction to alcohol can be considered as a disease or sign of other diseases; similarly, hypertension can be a disease (essential hypertension) or a sign of another disease (secondary hypertension). The same problem arises when we attempt to quantify a symptom as a medical sign; e.g., it is easy to assess the blood pressure of a patient with no ambiguity, whereas assigning an objective value to a symptom such as “feeling dizzy” is very difficult, considering that what one person means by saying that he or she feels dizzy does not necessarily match what another person means. In the following, however, we shall assume that symptoms can be somehow mapped to sign values and discuss only the signs, which in general could be multivalued or continuous variables. We note that very subjective symptoms can also be regarded simply as features or manifestations of a disease state. From a statistical physics point of view, it is natural to define all microscopic variables of a system as the signs and define the healthy and disease states of the system as the emergent or macroscopic behaviors of the system [48]. In the example of a biochemical reaction network, the number of molecules can be regarded as the microscopic variables (signs), whose stochastic dynamics are governed by the biochemical reactions [15,49]. Here, the system defects are defined as specific deviations from the healthy network, e.g., variations in the reaction rates. A subset of such defects could then result in a new macroscopic state (a disease), which manifests in the collective behavior of the molecular species. More precisely, the stochastic sign variables S are described by a probability distribution $P_{t}\left(S\right)$, which, in general, depends on the time *t*. This measure represents the uncertainties of the sign variables in a large ensemble of subjects. In practice, such a probability distribution (probabilistic model) can be reconstructed from the empirical data within a time period that is much shorter than the time scale of the system, and by relying on the maximum entropy principle [50]. Then, we may define a healthy or disease state as a (pure or mixed) Gibbs state of the sign probability distribution [51]. A pure Gibbs state is a macroscopic state of the probability distribution where linear correlations between the variables decay exponentially fast by the distance of the variables. A healthy system may display many healthy macroscopic states as a mixture of pure Gibbs states. In the same way, we may need mixed Gibbs states to describe the statistical behavior of a diseased system as for a glassy state [52].

Several scenarios are possible in the process of disease development as $P_{t}\left(S\right)$ changes with time [53]. A disease state may appear through: (i) A smooth change in the average sign values with no phase transition, e.g., as in aging. Here the system performance is degraded and the macroscopic state of the system is changing without any singularity in the system behavior. (ii) Discontinuous (sharp) phase transition—e.g., when the stress exceeds a critical value [54]. This happens when a metastable state gradually appears away from the main state and later dominates the system’s macroscopic behavior. On the other hand, we may consider a metastable state as the healthy state which disappears through a sharp transition to the stable macroscopic state (disease state). (iii) A continuous phase transition—e.g., when the strength of internal interactions between the sign variables increases [54]. These latter transitions can be further classified by the critical behavior of the system around the phase transition [55]. In the above picture, a disease state is characterized by the macroscopic behavior of the associated Gibbs state(s), e.g., by the structure and values of the order parameters that are needed to represent the (quasi)long-range order of the system. In addition, this picture provides a framework for classifying diseases in accordance with the nature of the phase transitions and the critical behaviors that are displayed during the process of disease development over time.

Artificial neural networks and machine learning techniques have been successfully employed to represent and distinguish the macroscopic states of various physical systems displaying various complex (including topological) phases [56,57,58]. This is very similar in spirit to the problem of identifying healthy and disease states, as described above. To summarize, taking the microscopic variables of the system as the signs, the problem of defining the diseases reduces to the problem of identifying and characterizing the Gibbs states of the signs probability distribution $P_{t}\left(S\right)$. Here, unsupervised (or partly supervised) learning approaches are needed for an accurate characterizations of disease phenotypes. Note that, in reality, we might only have access to a finite number of sign configurations which are sampled from a heterogeneous population of subjects possibly at different stages of disease development [59,60].

## 3. The Need for Deeper Probabilistic Models

It is known that an effect could have multiple causes and a cause may contribute to multiple effects. Moreover, there are no certain relations connecting a small subset of observed signs to a single (or multiple) disease(s). The early models of signs and diseases were based on specific rules connecting a piece of evidence to a hypothesis. Each rule was assigned a certainty factor to represent the experts’ belief on that rule, along with simple combination functions to compute the certainty factors for the composite rules [61]. However, the errors in these models arise if multiple causes are at work, especially when these causes are correlated [22].

The sign–disease dependencies can also be represented by a weighted graph of signs and diseases, to have a global view of the connectivity pattern of these variables. A complex network approach to the problem utilizes the structural and dynamical information extracted from the multiplex network of diseases, signs, proteins, etc., to reach a diagnosis [62,63,64,65,66]. The structural and functional modules of these networks provide a useful tool for classification of complex diseases, and from this information we can say something about the involving diseases given the observed signs [34]. The main focus in this approach is on the accurate construction of the above networks from the available clinical and biomedical data to reach a reliable diagnosis.

**Figure 2:**
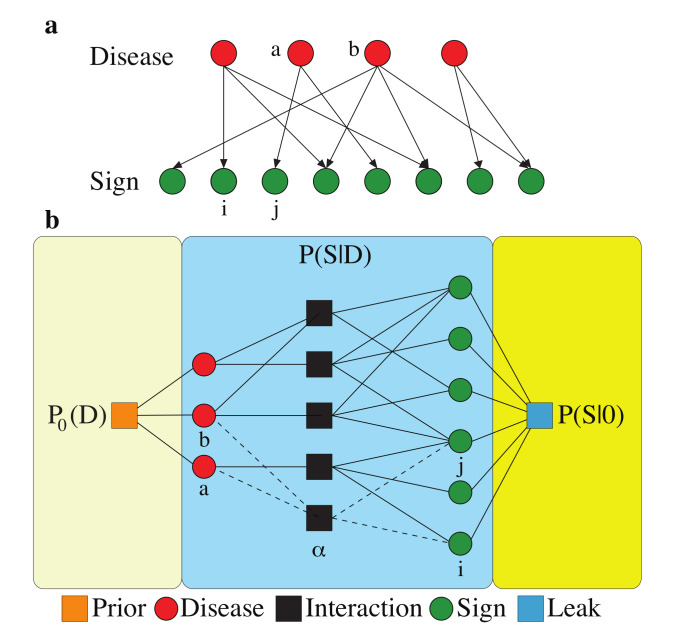
Probabilistic models of sign and disease variables: (**a**) A Bayesian belief network in the form of an acyclic directed graph showing the connections between the disease $\left(D_{a},D_{b},...\right)$ and sign $\left(S_{i},S_{j},...\right)$ variables. The model is completed by the conditional probability distribution P(S|D). (**b**) An interaction graph of disease variables (leftmost circles) and sign variables (rightmost circles) related by $M_{a}$ one-disease and $M_{ab}$ two-disease interaction factors (middle squares) in addition to interactions induced by the leak probability (right square) and the prior probability of disease (left square). (Adapted from reference [73]).

A complete description of the stochastic sign/disease variables, however, is provided by a joint probability distribution of these variables assuming that we have well-defined signs and diseases (see Section 2). Here, insights from the statistical physics of disordered systems could be useful in model construction and in the approximate inference of the local (microscopic) and global (macroscopic) statistical properties of such a model. Probabilistic models, e.g., Bayesian belief networks (Figure 2), allow us to model and account for the uncertainties in sign–disease relations more explicitly and accurately [67,68,69,70,71,72]. A belief network of the signs and diseases is an acyclic directed graph of the variables (without any hidden variable). The joint probability distribution of the variables P(S,D) in such a simple belief network is completely determined by the conditional probabilities of the child variables given the parents’ configuration.

To make the computations tractable in the above networks, it is commonly assumed that (i) given a disease hypothesis, the signs are independent stochastic variables. That is, the two sets of variables make a directed bipartite graph and we need to know only the conditional probabilities of the sign variables $P(S_{i}|\left\{D_{a}:a\in \pi \left(i\right)\right\})$ given the diseases in the parent set π(i). Moreover, it is assumed that (ii) the diseases are independent of each other after marginalization over the sign variables, i.e., $P\left(D\right)=\prod_{a}P\left(D_{a}\right)$. More importantly, it is also assumed that (iii) each disease affects the signs independently of any other diseases (causal independence) [21]. However, the signs could be strongly correlated even for a given disease hypothesis, and diseases are expected to interact with each other with potentially significant correlations. These correlations could be very helpful for facilitating early and more accurate diagnosis, especially in the presence of multiple interacting diseases. This encourages one to study deeper probabilistic models that also include disease–disease and sign–sign interactions (see Figure 2). For instance, given the one- and two-sign correlations for disease patterns D, the maximum-entropy probability distribution is $P\left(S|D\right)=\exp(\sum_{i}h_{i}\left(D\right)S_{i}+\sum_{\left(ij\right)}J_{ij}\left(D\right)S_{i}S_{j})/Z\left[D\right]$. Now, the model parameters $h_{i}\left(D\right),J_{ij}\left(D\right)$ can be expanded in terms of the disease variables $D_{a}$ to write the conditional probability in terms of a few disease interaction factors. This expansion should work when the number of diseases involved is expected to be small [73]. Considering at most the two-disease interaction factors, one gets

(1) $$\begin{array}{c} P\left(S|D\right)=\frac{1}{Z[D]}\phi_{0}\left(S\right)\prod_{a}\phi_{a}\left(S|D_{a}\right)\prod_{\left(ab\right)}\phi_{ab}\left(S|D_{a},D_{b}\right). \end{array}$$

This model, along with the prior probability of diseases $P_{0}\left(D\right)$, then identifies the joint probability distribution of the sign and disease variables. Deep belief networks provide another way of accounting for these interactions implicitly but a probabilistic model with explicit interaction factors would also allow for meaningful clinical interpretations [74,75,76].

It is not difficult to see that such interacting models can be used to estimate disease probabilities that closely follow the expected probabilities in the presence of strong sign–sign and disease–disease correlations [73]. In particular, such information could be very helpful for reaching a correct diagnosis, especially in the early stages of a disease. The clinical data that are needed here to construct the models are the joint probability distribution of two signs, $P(S_{i},S_{j}|D_{a},D_{b})$, conditioned on the presence of at most two diseases, $D_{a}$ and $D_{b}$. The apparent overparametrization may complicate the learning process and increase the risk of overfitting, but at the same time, it allows us to capture the essential features that are relevant to the problem [77,78,79]. Moreover, careful design of the model structure and algorithms could mitigate these difficulties, for example, by exploiting the power of generalized mean-field approximations and message-passing algorithms developed in the study of probabilistic graphical models [26,35,36,46]. Here, biomedical and computational insights are very helpful to start with as prior information to avoid the unnecessary model complexities. The computational cost of constructing and inferring from such models, and the lack of sufficient data, should of course be addressed if we are to benefit from these statistical correlations. This situation motivates us to develop more efficient and accurate learning and inference algorithms and justifies the collection of the relevant statistical data.

## 4. Search for an Optimal Diagnostic Strategy

In a clinical setting, a diagnostic problem is typically a multistage problem, where we start from a small set of initial findings and proceed by a sequence of hypothesis selection and testing [80,81,82,83]. For simplicity, we may assume that the duration of this diagnostic process is much less than the dynamical time scale of disease progress; i.e., the parameters of the probabilistic sign–disease model are fixed during the diagnostic process. Given an initial number of observed signs $N_{O}$, a key question is how to choose an optimal sequence O(T) of *T* other signs for observation (e.g., by maximizing an appropriate objective function). A classical choice here is the sequence that maximizes the likelihood of the most likely disease hypothesis after observation of the signs in the sequence [50]. A computationally simpler objective function E[O(T)] looks for a sequence which results in the largest polarization of the disease probabilities [84], considering also the diseases importance values:

(2) $$\begin{array}{c} E\left[O\left(T\right)\right]=\sum_{a}w_{a}\left|P\left(D_{a}\right)-\frac{1}{2}\right|. \end{array}$$

**Figure 3:**
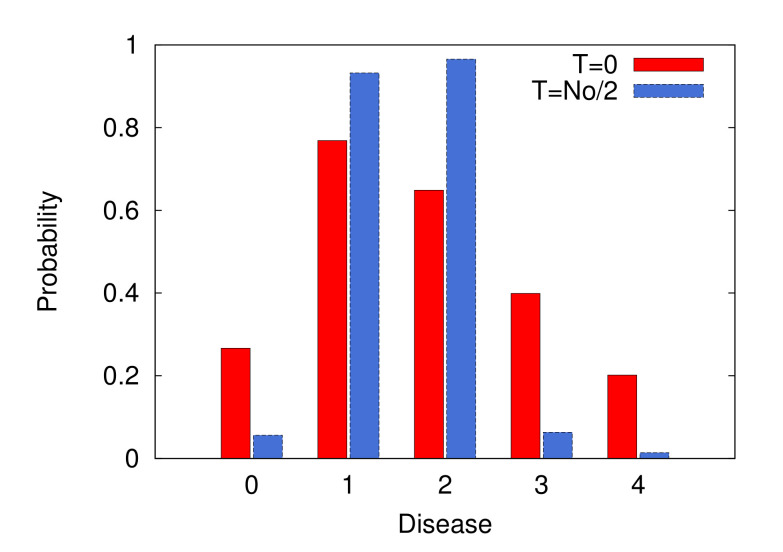
The impact of observing the most polarizing signs on the disease probabilities. The numbers of signs and diseases in this example are $N_{S}=20$ and $N_{D}=5$, respectively. The probabilistic sign–disease model is constructed by using synthetic conditional probabilities $P(S_{i}|D_{a})$ and $P(S_{i}|D_{a},D_{b})$ that are concentrated around the sign values randomly assigned to the diseases. The disease probabilities are computed exactly by an exhaustive algorithm (more details can be found in reference [84]). Given the $N_{O}=4$ initially observed signs, the algorithm anticipates the values of *T* other signs that would make the disease probabilities more decisive (closer to zero or one).

**Figure 4:**
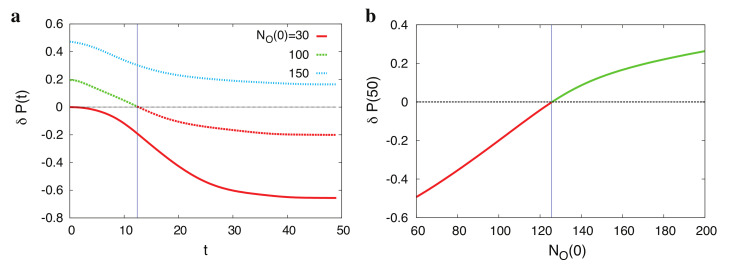
How the initial number of observed signs determines the range of useful predictions with a probabilistic model. (**a**) The difference $\delta P\left(t\right)=P\left(T_{R}\leq t\right)-P\left(T_{W}\leq t\right)$ between the cumulative probabilities of the first correct and incorrect diagnosis times ($T_{R}$ and $T_{W}$, respectively) is plotted against the number of observations *t* for different numbers of initial observations, $N_{O}\left(0\right)$. (**b**) δP(50) is plotted against $N_{O}\left(0\right)$ for a sufficiently large value of *t*. The numbers of signs and diseases in this example are $N_{S}=500$ and $N_{D}=50$. (Adapted from reference [84]).

Obviously, disease probabilities that are closer to zero or one allow us to reach a more definitive diagnosis. Other measures, e.g., the cost of observations or availability of the tests, may be added to this objective function. Figure 3 shows how this strategy increases the probability gap between the underlying diseases and the other diseases in a small synthetic example. Note that here, we are indeed simulating the diagnostic process using a probabilistic model of the signs and diseases. It should be mentioned that the usefulness of such a simulation critically depends on the structure of the probabilistic model and the initial number of observed signs. For reference, Figure 4 shows the differences in the probabilities of correct and incorrect diagnoses calculated by anticipating the values of a sequence of randomly selected signs, starting from $N_{O}\left(0\right)$ observed signs. Improvements in the model predictions are observed when the sequence of signs is suggested by the probabilistic model [84].

**Figure 5:**
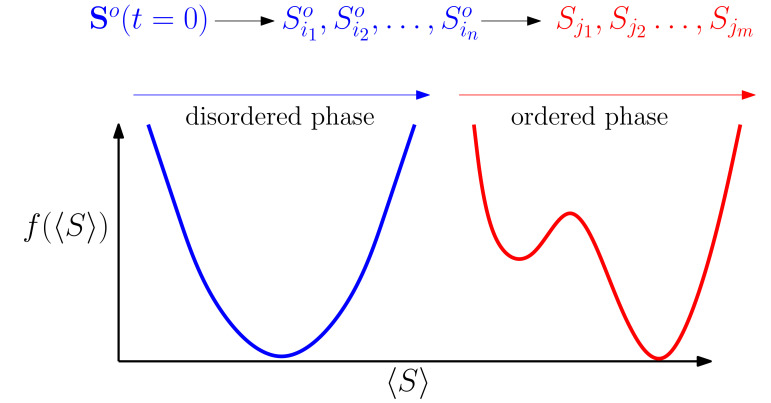
A diagnostic process that starts with the step-by-step approach and then switches to the batch approach after a phase transition to an ordered phase. In the disordered phase, the probability distribution of the signs is described by a single pure Gibbs state in which the observed signs on average give no information about the values of the unobserved signs. More observations can lead to a phase transition to an ordered phase in which there are multiple pure Gibbs states that provide useful information about the unobserved signs.

The main finding here is the advantage of a two-stage diagnostic strategy [85], which starts with suggesting one medical test in each step and observing the outcome of that medical test (Figure 5). Then, at a critical number of observations, the probabilistic model undergoes a phase transition to an ordered phase in which it is safe to suggest a sequence of several medical tests at once based on the model predictions. A similar phenomenon is observed in the “ordered” phase of a physical system where the boundary or pinned variables can strongly affect the state of other distant variables [86,87]. The above studies show that it is possible to obtain useful information by simulating a diagnostic process using sign and disease probabilities inferred from a reasonable probabilistic model. Note that the above problem is indeed a stochastic optimization problem. This is because, at each point in sequence O(T), we have only the sign probabilities estimated from the model without any real medical test having been performed. More precisely, the objective function E[O(T)] depends on the observed sign values $S_{O}$ in the sequence. Therefore, in order to find the optimal sequence, the right objective function is 〈E[O(T)]〉, where the average is taken over the observed sign values. The established techniques of stochastic optimization are needed here to accurately investigate the problem [88,89,90].

## 5. Future Perspectives

The future technological advancements are going to revolutionize the way diagnosis is going to be done [91,92]. There are already some AI systems in use, such as IBM’s Watson or Babylon’s AI chatbot. These approaches are still at their infancy and do not consider the complexity of human biology and the real-world diagnostic problems. Handling additional complexity requires innovative algorithms. In this perspective article we gave a glimpse into what statistical physics can contribute in this regard. A major challenge that prevents implementation of these physics-based algorithms (and more generally, all currently existing AI algorithms) is a lack of rigorous clinical validation. Due to these limitations, current use of AI in medicine is mostly limited to interpretation of medical images and the like. This problem will eventually be solved in the years to come. Temporal data from healthy and diseased individuals will be available. Wearable and portable devices such as watches or smartphones, are now able to monitor our health round the clock (e.g., pulse rate, blood pressure, ECG). Probes will be developed that allow for continual sensing of biological contents in our sweat, saliva, urine, and stool. Longitudinal cohorts on large populations will provide clinical and laboratory data needed to build and validate the models discussed above.

Many opportunities for medical diagnostics lie at the interface of physics and AI. In this article, we have briefly discussed the state of the art in this direction of study, but there are many more possibilities to be explored in the future, for example, by including disease dynamics or by benefiting from developments in quantum-physics-based approaches.

### 5.1. Diagnosis through Simulation of Disease Evolution

Obviously, availability of tractable microscopic models for the temporal evolution of diseases would be significantly helpful in addressing the diagnostic problems. Currently, we lack such models for most of the common diseases, mainly because of the lack of relevant clinical and experimental data, or maybe because diseases are usually considered as static objects. Some small steps have been taken though and efforts have been made to represent disease progression at different spatial and temporal scales [93,94,95,96]. For instance, models exist that incorporate molecular processes involved in diseases [97]. In contrast, the methods of ecological and resource-consumer theory are used to study tumor growth dynamics and the host-pathogen interactions at the level of cell populations [98,99]. At a larger scale, complex system approaches are applied to model the dynamics of neurological disorders [100]. Currently, our understanding of dynamics of diseases is often minimal and insufficient. Acquisition of temporal clinical data and monitoring of diseases dynamics are critical here for understanding of disease development [101,102]. The methods of complex dynamic systems and machine learning can then be employed to analyze the data and construct reasonable dynamical models.

A stochastic model for disease evolution could be very useful for generating a diagnosis that is based on the history (dynamics) of the sign variables. Consider again a biochemical reaction network and assume that the system starts from a healthy state that maximizes an objective function of the system, e.g., the mutual information between a subset of signal species and a subset of response species. A model for disease evolution should describe the emergence of other macroscopic states in terms of the changing number and strengths of the possible defects in the system. A minimal effective model here is described by two kind of parameters, say $\alpha_{r}\left(t\right)$ and 1/β(t), which control the rate of introducing local defects or mutations that affect reaction *r* in the network and the rate of accepting these variations by the global system (e.g., defense mechanisms such as the immune system or intracellular quality control systems), respectively [53]. Disease progress then is modeled by a reverse annealing process, where both the above rates may increase with time starting from the healthy state. This model is inspired by the thermal annealing of physical systems as in the simulated annealing algorithm, where the temperature is slightly reduced to bring the system to an ordered low-temperature state, starting from a disordered high-temperature phase [103]. Here, in contrast, we are using a reverse annealing algorithm to model disease evolution by increasing the temperature-like parameter 1/β(t), to go from an optimal healthy state (say lower energies) to a disease state with smaller objective functions (or larger energies).

Now suppose that we have observed a subset of the molecular concentrations over a sufficiently long interval of time. A relevant problem then is to reconstruct the time evolution of the model parameters $\alpha_{r}\left(t\right),\beta \left(t\right)$ to identify the underlying defects and the disease(s) to which they can be attributed. Clearly, a diagnosis that relies on the likelihoods of diseases over a given time history would be more accurate than a diagnosis that is based solely on the current sign values. We observed in the previous section that simulating the diagnostic process is helpful for suggesting an optimal sequence of medical tests given an initial number of observed signs. For this purpose, we needed a good probabilistic model in order to infer the sign and disease probabilities in each step of simulation. For diagnosis based on dynamics, we need a good microscopic model for simulating the evolution of diseases over time and considering the possible disease–disease interactions [53]. Such a model would also allow us to see how the accuracy of diagnosis with a diagnostic algorithm depends on the elapsed time of disease progression. On one hand, we know that disease morbidity and mortality often increase with time and the chance of successful therapy decreases; thus, clinicians often strive for an early diagnosis. A diagnostic algorithm should then come with low probabilities of false positive and false negative results to avoid the negative consequences of a wrong early diagnosis. On the other hand, the diagnosis accuracy is expected to increase with time as the observed signs would convey more information about the underlying disease(s). The above information are necessary for an accurate quantification of the tradeoff between the accuracy and timing of diagnosis, thus enabling the identification of an optimal intervention time.

A microscopic model of disease evolution is also useful for comparing the evidence supporting different hypotheses through simulation when the likelihood function is difficult to compute, that is, for a likelihood-free estimation of evidence such as the approximate Bayesian computation (ABC) method [104,105,106,107]. Simulation-based methods of this kind are now well established in the physical sciences, e.g., in experimental particle physics and cosmology [56]. Moreover, such a microscopic model can play the role of a discriminative model as the counterpart to the generative probabilistic model in an adversarial process [108,109,110,111]. Finally, all of the above models and studies can be personalized within the framework of precision medicine.

### 5.2. Quantum Algorithms

Quantum systems, in contrast to classical systems, are described by a superposition of microscopic states and display nonlocal quantum correlations (entanglement). These nonclassical behaviors can be exploited by quantum computers and algorithms to reduce the time and memory complexity of computationally hard problems [112]. Advances in classical and quantum machine learning techniques and algorithms are very promising in finding approximate solutions to such problems. In particular, steady progress in quantum computation technology encourages us to apply and extend the above quantum algorithms to the computationally difficult and important problem of medical diagnostics. Quantum representations can be useful even within a classical computation [113,114,115,116,117]. For instance, an exponentially large number of classical states can be coded in the quantum state or wave function of a linear number of quantum binary systems (qbits). Quantum wave functions also provide a rich class of variational probability distributions that can be used to approximate the macroscopic states of classical stochastic variables. There are examples of variational wave functions from physics, for example constructed by neural networks, which can provide good probabilistic models for the sign and disease variables. A quantum learning algorithm here is needed to find the optimal parameters of the wave function. Building on this, it would be interesting to see whether quantum representations of the probabilistic models of the sign and disease variables would be helpful in solving a diagnostic problem. On the other hand, the log-likelihood of a disease hypothesis in a Bayesian belief network (with the three simplifying assumptions of Section 3) can be considered as the energy function of a classical system with local interactions between the disease variables. This problem can then be studied by using quantum optimization techniques (e.g., quantum annealing algorithms or quantum machine learning methods) to exploit the computational power of quantum representations and systems [118,119,120,121].

## 6. Conclusions and Challenges

In summary, it seems that more accurate definitions of the signs/symptoms and diseases involved in a diagnostic problem are needed for the precise characterization of the statistical relationships and possible interactions between these variables. The problem here is to choose the relevant signs/symptoms as the microscopic variables of the system and find informative order parameters to characterize the macroscopic or emergent features of this interacting system as the disease states. The above definitions, in turn, would allow us to construct better (deep) probabilistic models of the signs and diseases, which would play a critical role in enabling early diagnosis, for example, through the simulation of the diagnostic process, as described above. The main challenge is to make a balance between the model efficiency and its predictability (generalization), and interpretability. Obviously, to gain from such models, we need to invest in collecting the necessary clinical data and in developing more efficient and accurate inference and learning algorithms. The point is that collecting good higher order statistical data is very difficult in practice, even for two-sign probability distributions conditioned on the presence of one or two diseases $P(S_{i},S_{j}|D_{a},D_{b})$.

As another approach, we may incorporate the time dimension into diagnostic problems to benefit from the dynamical information provided by the history of the observed signs. For instance, it may happen that the observed signs give the same probabilities for two diseases when we work with a static sign–disease model. A way out of this could of course be to enlarge the space of sign variables to discriminate the two cases. On the other hand, one may look at the history or time dependence of the observed signs to reach a diagnosis that is based on the dynamics of diseases. A microscopic model of the temporal evolution of diseases is needed here to infer the underlying diseases through the simulation of the stochastic disease dynamics considering also the relevant host factors in the prior information. In addition, it can be used for explicit modeling of disease–disease interactions. Here, the main problem is construction of biologically plausible models which help us to quantify the changes in the potential risk of diseases with time. This information, along with the knowledge of diagnosis accuracy as a function of time, helps one to decide on intervention options, and to avoid over screening. Finally, we may consider the possibility of mitigating the computational complexity of diagnostic problems by utilizing the computational power of quantum optimization and learning algorithms. 

## Figures and Tables

**Table 1:** An analogy between the main concepts of medical diagnostics and statistical physics.

Medical Diagnostics	Statistical Physics	Description
signs	microscopic variables	binary genotypes as two-state spins in a magnetic system
causal dependencies	Hamiltonian interactions	influencing factors as interactions with external fields and other spins
uncertainty and noise	temperature	stochastic variability from thermal fluctuations
healthy and disease states	thermodynamic phases	emergent phenotypes as macroscopic features of Gibbs states
observed signs	pinned microscopic variables	related to random pinning transitions
diagnosis	phase detection	similar to the phase classification problem
